# The Transition of Urological Practice for Forty Years in Juntendo University

**DOI:** 10.14789/jmj.JMJ22-0027-R

**Published:** 2022-10-04

**Authors:** YOSHIRO SAKAMOTO

**Affiliations:** 1 Department of Urology, Juntendo University Nerima Hospital, Tokyo, Japan; 1 Department of Urology, Juntendo University Nerima Hospital, Tokyo, Japan

**Keywords:** prostate cancer, transperineal prostate biopsy, robot-assisted radical prostatectomy

## Abstract

I enrolled in the Juntendo University Urological Course in 1982 and worked at Juntendo for 40 years until I retired in 2022. The transition of Urology had been slow until recent years. In the last 20-30 years, Urological practice has made significant progress. I will look back on the 40 years of Juntendo University and describe it in this article. In particular, the transition and breakthrough in the diagnosis and treatment of prostate cancer were remarkable. This point will be described mainly based on the experience at Juntendo Nerima Hospital.

In Japan, in the early Meiji era, German medicine was required to have the norms of modern medicine, and Urology was introduced along with Dermatology in the early 30th year of the Meiji era. Therefore, Urology in Japan has a strong connection with Dermatology until after World War II, and the course has continued as a Dermatology and Urology class at each medical education institution. On the other hand, in the United States, the Urology was the department of surgery, division of Urology. At the dawn of Juntendo University, the Department of Urology discharged very talented and well-known urologists.

## Juntendo Urology Faculty Part-1

1901 (Meiji 34) Faculty of Juntendo: Susumu Sato (Surgery), Tatsujiro Sato (Surgery), Saburo Akutsu (Urology)

**Figure 1:**
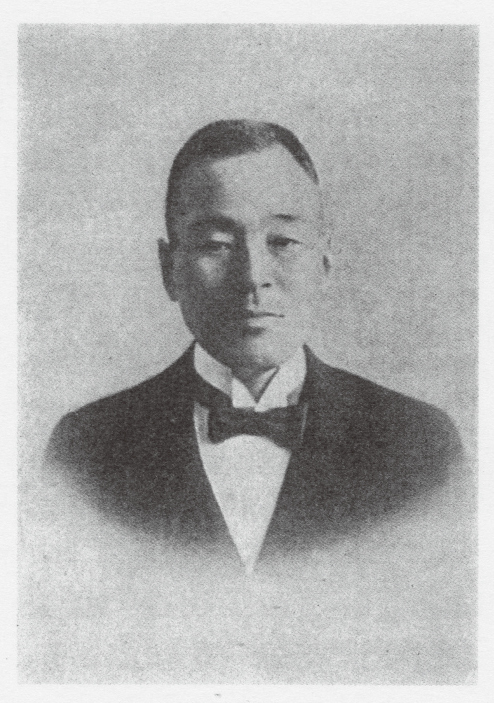
Prof. Saburo Akutsu

Prof. Saburo Akutsu (Figure 1) studied Urology at the University of Vienna and was one of the pioneers of Japanese Urology.

**Figure 2:**
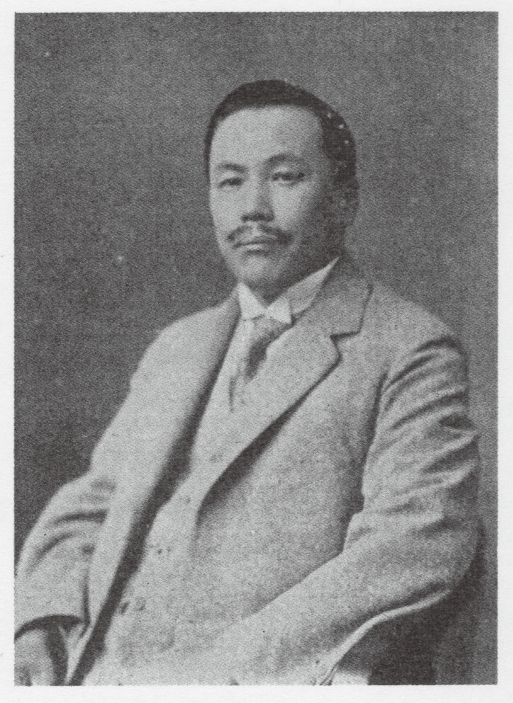
Prof. Isamu Sakaguchi

1908 (Meiji 41) Prof. Isamu Sakaguchi (Figure 2) opened Dermatology.

1915 (Taisho 4) Dr. Sakaguchi became a professor of Dermatology and Urology, and later became a full-time in Urology. He made an effort in the domestic production of cystoscopes. In recognition of his achievements, the Sakaguchi Award was established at the Japanese Urological Association.

## Juntendo Urology Faculty Part-2

1943 （Showa 18） Prof. Masatoki Koike

1958 （Showa 33） Prof. HiromotoTakahashi

1982 （Showa 57） Prof. Ryuichi Kitagawa

1993 （Heisei 5） Prof. Makoto Fujime

2012 （Heisei 24） Prof. Shigeo Horie

## The Transition of Urology for forty years

**Figure 3:**
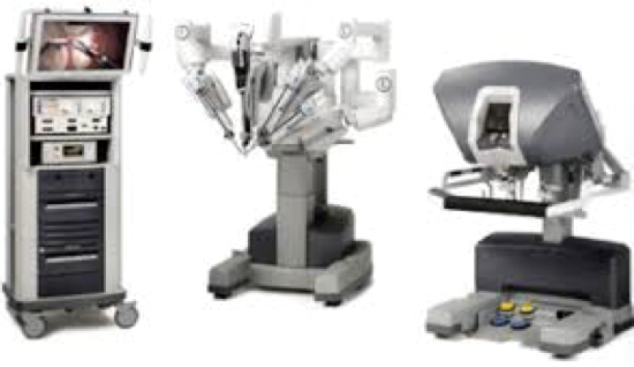
Intuitive Da Vinci surgical system

The prostate cancer practice has undergone significant changes over the last 40 years. Forty years ago, when I was still a new physician, surgical castration was the first operation for a new urologist. At that time, surgical castration was the only surgery for prostate cancer and was the mainstay of hormone therapy for prostate cancer. Currently, robot-assisted radical prostatectomy (RARP, Figure 3) has become a main surgical procedure that is widely performed not only in Western countries but also in Japan. Hormone therapy for prostate cancer has also started to use new AR target drugs (Abiraterone, Enzalutamide, Apalutamide, and Darolutamide) as well as surgical castration and medical castration. In addition, anticancer drugs such as Docetaxel and Cabazitaxel have been used, and radiation therapy for bone metastases such as Ra-223 has also been administered.

**Figure 4:**
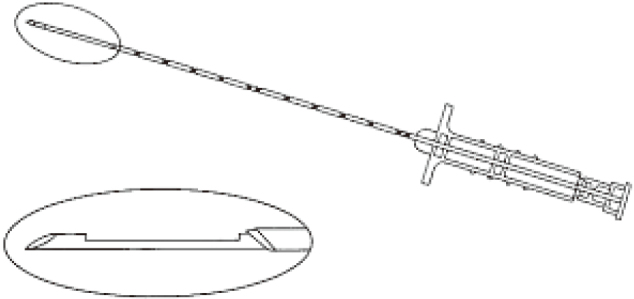
TruCut needle

**Figure 5:**
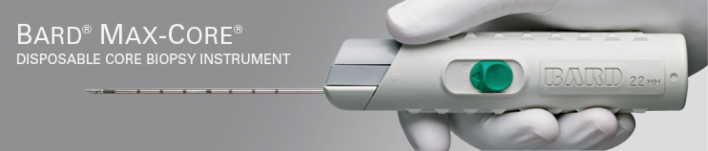
BARD^®^　MAX-CORE^®^

Prostate biopsy has also changed significantly. In the 1980s, when induration of prostate cancer was palpable, prostate biopsy was performed using a TruCut needle (Figure 4) under a finger guidance. In recent years, puncture needles have also advanced, allowing us to use superior products (Figure 5). PSA measurement became possible by Wang et al.^1)^ in 1979, Holm et al.^2)^ performed a transrectal ultrasound-guided prostate biopsy (TRUS- B) in 1981.

**Figure 6:**
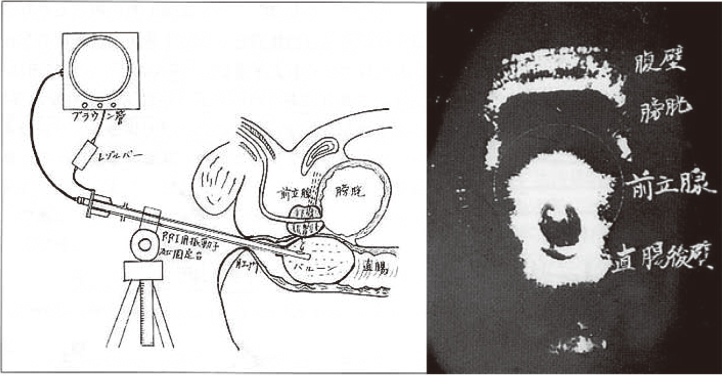
Transrectal prostate ultrasound

In Japan, in 1963, Hiroki Watanabe of the Department of Urology, Kyoto Prefectural University of Medicine, Tatsuo Ouchi, Hiromoto Takahashi, and Toshio Wagai of Juntendo University developed and clinically applied transrectal prostate ultrasound (Figure 6).

**Figure 7:**
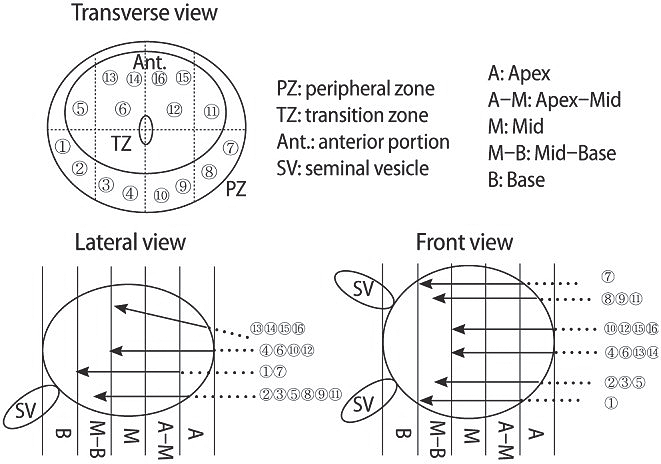
Transperineal prostate biopsy : transverse view, lateral view, and front view

**Figure 8:**
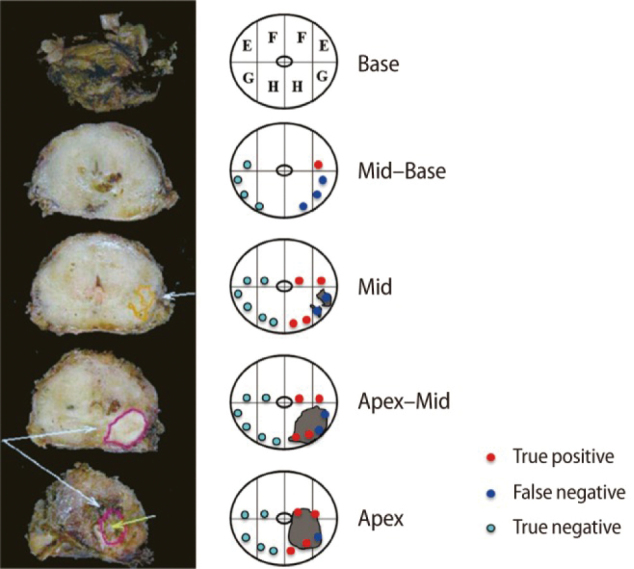
Transverse view : prostate cancer localization and transperineal prostate biopsy

Analysis of prostate cancer localization toward improved diagnostic accuracy of transperineal prostate biopsy^3)^. Delineating the precise localization of prostate cancer is important in improving the diagnostic accuracy of prostate biopsy. In Juntendo University Nerima Hospital, initial 12- core or repeat 16-core biopsies were performed using a transrectal ultrasound guided transperineal prostate biopsy method. We step-sectioned prostates from radical prostatectomy specimens at 5-mm intervals from the urethra to the urinary bladder and designated five regions: the (1) Apex, (2) Apex-Mid, (3) Mid, (4) Mid-Base, and (5) Base. We then mapped prostate cancer localization on eight zones around the urethra for each of those regions. Prostate cancer was detected in 93 cases of 121 cases (76.9%) in the Apex, in 115 cases (95.0%) in the Apex-Mid, in 101 cases (83.5%) in the Mid, in 71 cases (58.7%) in the Mid-Base, and in 23 cases (19.0%) in the Base. In 99.2% of all cases, prostate cancers were detected from the Apex to Mid regions. For this reason, transperineal prostate biopsies have routinely been prioritized in the Apex, Apex-Mid, and Mid regions, while the Base region of the prostate was considered to be of lesser importance. Our analyses of prostate cancer localization revealed a higher rate of cancer in the posterior portion of the Apex, antero-medial and postero-medial portion of the Apex-Mid and antero-medial and postero-lateral portion of the Mid. The transperineal prostate biopsies in our institute performed had a sensitivity of 70.9%, a specificity of 96.6%, a positive predictive value (PPV) of 92.2% and a negative predictive value (NPV) of 85.5% (Figure 7, 8, Table 1, 2).

**Table 1:** Prostate cancer localization in the Apex, Apex–Mid, Mid, Mid–Base, and Base out of the 121 cases

Region	No. of cases of prostate cancer positive (%)
Apex	93 (76.9)
Apex–Mid	115 (95.0)
Mid	101 (83.5)
Mid–Base	71 (58.7)
Base	23 (19.0)

Ninety-nine point two percent of all cancer cases were detected in the Apex and Apex–Mid regions, 95.5% was detected in the Apex–Mid and Mid regions, and 84.3% was detected in the Mid and Mid–Base regions.

**Table 2:** Concordance of prostate cancer in prostatectomy specimen and biopsy

	Prostate cancer (+)	Prostate cancer (−)	Total
Biopsy (+)	344	29	373
Biopsy (−)	141	834	975
Total	485	863	1,348

Sensitivity (=TP/[TP+FN]), 70.9%; specificity (=TN/[FP+TN]), 96.6%; positive predictive value (=TP/[TP+FP]), 92.2%; negative predictive value (=TN/[TN+FN]), 85.5%. True positive (TP)　False positive (FP) False negative (FN)　True negative (TN)

## Conclusions

The concordance of prostate cancer between prostatectomy specimens and biopsies is comparatively favorable. According to our study, the diagnostic accuracy of transperineal prostate biopsy can be improved in our institute by including the anterior portion of the Apex-Mid and Mid regions in the 12-core biopsy or 16-core biopsy, such that a 4-core biopsy of the anterior portion is included.

**Figure 9:**
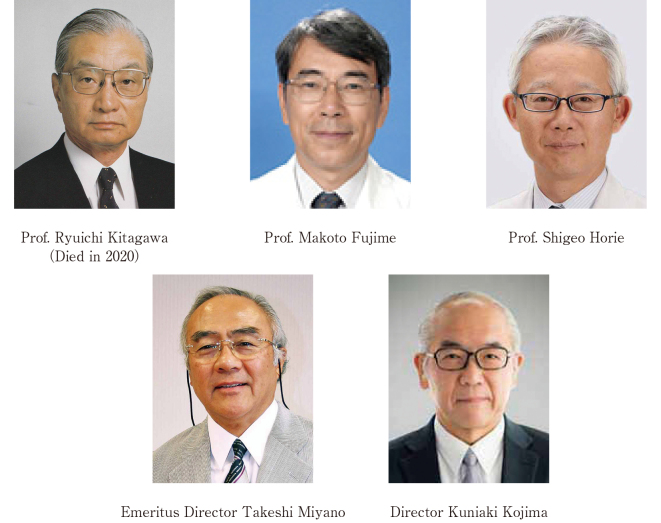
Three chief professors and two directors who were my mentors

During my 40 years at Juntendo University, I served three chief professors and two directors (Figure 9). Thanks to these doctors, I was able to carry out a very fulfilling and happy medical practice. We would like to express our sincere gratitude to all the bosses and staff who have been involved in reaching retirement age.

## Funding

Not applicable.

## Author contributions

YS contributed to drafting the manuscript, figures and tables.

## Conflicts of interest statement

The author declares that there are no conflicts of interest.
